# Correction: Renteria-Flores et al. Transforming Properties of E6/E7 Oncogenes from Beta-2 HPV80 in Primary Human Fibroblasts. *Int. J. Mol. Sci.* 2025, *26*, 5347

**DOI:** 10.3390/ijms27052176

**Published:** 2026-02-26

**Authors:** Francisco Israel Renteria-Flores, Andrea Molina-Pineda, Ruben Piña-Cruz, Sayma Vizcarra-Ramos, Alejandra Natali Vega-Magaña, Mariel García-Chagollán, María Teresa Magaña-Torres, Rodolfo Hernández-Gutiérrez, Adriana Aguilar-Lemarroy, Luis Felipe Jave-Suárez

**Affiliations:** 1 Programa de Doctorado en Ciencias en Biología Molecular y Medicina, Centro Universitario de Ciencias de la Salud (CUCS), Universidad de Guadalajara, Guadalajara 44340, Jalisco, Mexico; renteriaf33@gmail.com (F.I.R.-F.); ruben.pinacruz@gmail.com (R.P.-C.); 2 División de Inmunología, Centro de Investigación Biomédica de Occidente, Instituto Mexicano del Seguro Social (IMSS), Guadalajara 44340, Jalisco, Mexico; sayvimay0510@gmail.com; 3 Unidad de Biotecnología Médica y Farmacéutica, Centro de Investigación y Asistencia en Tecnología y Diseño del Estado de Jalisco A.C. (CIATEJ), Guadalajara 44270, Jalisco, Mexico; andymopi@gmail.com (A.M.-P.); rhgutierrez@ciatej.mx (R.H.-G.); 4 Programa de Doctorado en Ciencias Biomédicas, Centro Universitario de Ciencias de la Salud (CUCS), Universidad de Guadalajara, Guadalajara 44340, Jalisco, Mexico; 5 Laboratorio de Investigación en Cáncer e Infecciones, Centro Universitario de Ciencias de la Salud (CUCS), Universidad de Guadalajara, Guadalajara 44340, Jalisco, Mexico; alejandra.vega@academicos.udg.mx; 6 Instituto de Investigación en Ciencias Biomédicas, Centro Universitario de Ciencias de la Salud (CUCS), Universidad de Guadalajara, Guadalajara 44340, Jalisco, Mexico; chagollan@academicos.udg.mx; 7 División de Genética, Centro de Investigación Biomédica de Occidente, Instituto Mexicano del Seguro Social (IMSS), Guadalajara 44340, Jalisco, Mexico; maganamt@gmail.com

There was an error in the original publication [1]. A reference included in the published manuscript (cited as reference number 41), was retracted by the publisher in April 2025: “ABCG1 as a potential oncogene in lung cancer. doi:10.3892/etm.2017.4393. https://pubmed.ncbi.nlm.nih.gov/28588672/”. To uphold the highest standards of academic integrity and ensure the accuracy of the scientific record, the authors replaced the retracted reference with “The role of ATP-binding cassette subfamily G member 1 in tumor progression. Xu Xinyi; Yang Gong. Cancer Med. 2024. PMID: 38896016” [2].

A correction has been made to the Discussion, paragraph number 5:

As observed in Figure 6, the most significant downregulated gene in the E6/E7-HPV80 model includes *ABCG1*. The role of *ABCG1* in cancer is contradictory. Whereas it has been reported to promote tumorigenesis in some cancers, it exhibits an antiproliferative function in others [41]. This discrepancy suggests that the role of *ABCG1* in cancer may vary depending on the tumor type and cellular environment.

The authors state that the scientific conclusions are unaffected. This correction was approved by the Academic Editor. The original publication has also been updated.
